# Advancements in Laparoscopic Partial Nephrectomy: Expanding the Feasibility of Nephron-Sparing

**DOI:** 10.1155/2012/148952

**Published:** 2012-05-09

**Authors:** Eugene J. Pietzak, Thomas J. Guzzo

**Affiliations:** ^1^Division of Urology, Department of Surgery, Hospital of the University of Pennsylvania, Philadelphia, PA 19104, USA; ^2^Perelman Center for Advanced Medicine, University of Pennsylvania, 3400 Civic Center Boulevard, Philadelphia, PA 19104, USA

## Abstract

Partial nephrectomy (PN) offers equivalent oncologic outcomes to radical nephrectomy (RN) but has greater preservation of renal function and less risk of chronic kidney disease and cardiovascular disease. Laparoscopic PN remains underutilized likely because it is a technically challenging operation with higher rates of perioperative complications compared to open PN and laparoscopic RN. A review of the latest PN literature demonstrates that recent advancements in laparoscopic approaches, imaging modalities, ischemic mitigating strategies, renorrhaphy techniques, and hemostatic agents will likely allow greater utilization of LPN and expand its usage to increasingly more complex tumors.

## 1. Introduction

Partial Nephrectomy (PN) is the treatment of choice whenever feasible for enhancing renal masses [1–3]. For a multitude of reasons, PN has evolved from an operation performed in patients with an absolute indication for nephron sparing surgery (NSS) to avoid dialysis (solitary kidney, bilateral synchronous masses, or hereditary syndrome), to the preferred procedure for patients with renal masses <7 cm even with a normal contralateral kidney [4].

## 2. Rationale for a Partial Nephrectomy

 PN has proven to provide equivalent oncological outcomes to radical nephrectomy (RN) for renal tumors <4 cm (T1a) and, even more recently, tumors <7 cm (T1b) [5–9]. Multiple retrospective studies have shown no difference between PN and RN with regard to cancer-specific survival and rate to distant metastasis at long-term follow-up, but with greater renal function perseveration with PN [9–13]. Furthermore, several retrospective studies associate PN with better overall survival compared to RN [10, 14–17]. However, further investigation is still needed as the only prospective randomized trial comparing PN with RN showed an overall survival advantage for RN when using a intention-to-treat analysis [18].

 The risk of local recurrence with PN is also very low [19, 20]. Initial concern over recurrence within the tumor bed and the need for a wide resection margin, which may have been deterrents for selecting PN, have been shown to be unfounded as local recurrences after PN are rare and typically occur in the presence of grossly positive surgical margins [19–23]. Similarly, the risk for multiple tumor foci is less than 6% for T1a tumors [24]. With improved accuracy of both preoperative and intraoperative imaging techniques the concern of missing satellite tumor lesions in the ipsilateral kidney is unwarranted. Taken together, the lifetime risk of a new tumor developing in the ipsilateral kidney is less than 5% [19], similar to the risk for a tumor in the contra-lateral kidney [25].

 Furthermore, evidence continues to support the benefit of PN with regard to improved long-term renal and cardiovascular function [16, 26]. Arguments supporting RN having minimal impact on renal function have largely been extrapolated from literature on donor nephrectomy patients who are carefully screened and selected [27, 28]. Therefore, much of this evidence is not generalizable to the renal cell carcinoma population, who tend to be older and at greater risk for chronic kidney disease (CKD) and death [26].

With the increasing utilization of cross-sectional imaging for other reasons, there continues to be a shift toward smaller tumor size in patients with significant medical comorbidities, including renal insufficiency [26, 29, 30]. In fact, roughly 26% of patients presenting with renal masses <4 cm already have a baseline-estimated glomerular filtration rate (GFR) of <60 mL/min prior to surgery [31]. Examination of nontumor containing parenchyma of RN specimens has shown that the majority have some degree of renal histopathologic abnormalities [32]. Therefore, it is not surprising that RN is a risk factor for the development or worsening of chronic kidney disease (CKD) [31].

 Increasing attention has also been given to the link between CKD and cardiovascular disease (CVD) [33–35]. As GFR decreases, the risk for development of CVD increases as well [35]. Furthermore, the risk of both death and hospitalization increases as GFR drops below <60 mL/min [35, 36]. RN is a recognized risk factor for the development and progression to CKD [31], while preservation of nephrons from PN can mitigate these effects [10, 26, 31, 37, 38]. Furthermore, retrospective studies comparing PN to RN showed a reduction in CVD-related morbidity and mortality with PN [16, 17].

 Also it is important to realize that sufficient renal function is required for patients to receive many of the systemic medical therapies available in the event of recurrence [39]. With continued advancements in medical therapies, nephron sparing with PN may have an even greater advantages over RN.

## 3. Rationale for Laparoscopic Partial Nephrectomy

 Laparoscopic PN (LPN) is an acceptable alternative to open PN (OPN) for the treatment of T1 renal masses when performed by a skilled laparoscopic surgeon [1, 4], advances in surgical technique and the use of hemostatic agents have expanded the indications for LPN to more complex renal masses [40].

 LPN has equivalent oncologic outcomes to OPN, albeit with shorter follow-up, but offers the advantages of minimally invasive surgery [41, 42]. Several studies have demonstrated shorter length of hospitalization, quicker overall convalescence, lower narcotic requirements, improved cosmesis, and earlier diet resumption when compared to OPN [4, 43, 44]. The typically shorter length of hospital stay may also outweigh the usually longer surgical time of LPN to lead to a lower expense with LPN [45]. Although some experienced surgeons have been able minimize the morbidity of OPN to achieve similar convalescence of a minimally invasive surgery [46–48].

 Despite the benefits of LPN, it remains a technically challenging surgical procedure with a higher rate of intraoperative complications and longer warm ischemia time when compared to OPN [44, 49]. LPN has also been associated with increased rate of bleeding and urinary leak perioperatively [49, 50]. The longer ischemic time of LPN likely reflects the difficulty of renorrhaphy with laparoscopy. However, refinements in surgical technique and technological advances will likely lead to a reduction in complication rates, improved outcomes, and more widespread adaption of LPN [42].

## 4. Laparoscopic Approaches

 At this time, no good randomized data exists on the optimal technique for minimally invasive partial nephrectomy. The choice of approach is likely to be influenced by the individual surgeon's training and comfort level, as much as patient and tumor characteristics. Many of the minimally invasive techniques described often mimic the steps of an OPN [51].

 In general, the “pure” laparoscopic approach is the most challenging. Although excellent outcomes are possible in expert hands, the learning curve is steep [52]. A beginner to LPN likely should perform at least 10–20 small nonhilar lesions as their initial case experience [53]. At many community and regional hospitals the case volume may not be large enough to allow proficiency in LPN. In these situations, the approach which maximizes the chance for renal preservation and excellent oncologic control should be used [54]. For those with less laparoscopic experience, Hand Assisted Laparoscopy (HAL) may be a better option for LPN, as it has a quicker learning curve with reported improvements after only 4 cases [55, 56]. HAL PN also provides the additional benefit of allowing compression of renal parenchyma by hand, which can provide hemostasis without the need for vessel clamping [55]. If LPN is not feasible, most patients are better served by an OPN rather than a laparoscopic radical nephrectomy [16].

 Among the biggest advances in minimal invasive surgery is the introduction of robotic assistance laparoscopy. The benefits of 3D vision, 540-degree movement, and tremor elimination have been well described and there is a growing body of literature on the use of robotic-assistance in PN [57–59]. Robotic assisted laparoscopic PN (RALPN) also has a shorter learning curve than traditional LPN [57]. Although early reports have not shown lower complication rates or shorter ischemic time, with increasing experience this may change [52]. Some suggest that robotic assistance will allow an expansion of LPN to more difficult tumors, such as posterior, central, or hilar lesions [20, 60]. However, implementation and wide spread adoption of RALPN may be limited by the increased expense of RALPN over traditional LPN and OPN [61–63]. A recent meta-analysis revealed traditional LPN, at a mean direct cost of $10,311, more cost-effective than both RALPN at $11,962 and OPN $11,427 [62]. Future studies may reveal that improvements in clinical outcomes can compensate for the high costs of acquisition, maintenance, and disposable instruments involved with robotics [63]. This may only be feasible at centers with high robotic surgical volume [63].

 Another recent development in laparoscopic surgery is the concept of laparoendoscopic single-site surgery (LESS). Although still in its infancy for PN, successful cases have been reported with LESS [64]. The loss of instrument triangulation makes LESS difficult, but it is feasible in expert hands [65]. The use of robotic assistance with LESS may help facilitate this technique [65]. However, other than improved cosmesis, the benefits of LESS over other laparoscopic approaches are yet to be determined.

 Regardless of the laparoscopic approach initially chosen, if difficulty occurs during the operation, it is more appropriate to convert to an open or HAL approach if PN is still feasible, rather than a laparoscopic RN. The long-term benefits of nephron sparing surgery certainly outweigh the short-term advantages of minimally invasive approaches.

## 5. Novel Imaging

 A well-known drawback of laparoscopic surgery is the lack of tactile feedback it provides; this is particularly true with robotics. Although this is an area of active investigation, currently, this shortcoming increases the dependence on preoperative imaging and intraoperative visual cues [66].

 Preoperative modalities using 3D reconstructions of helical CT scans or MR angiography provide excellent views of the relationship of the tumor to the collecting system and renal vasculature [67]. This may help guide the intrarenal dissection to maximize the amount of parenchyma preserved while still achieving negative margins [66].

 Preoperative imaging also has an emerging role in diagnosing and determining need for therapy. In the current era, with the increasing proportion of incidentally found renal masses, a downward stage migration and concomitant increase in benign pathology has been well described [1, 26, 30]. Novel techniques using immunhistochemical or cytogenetics may better predict biological aggressiveness, which may help determine if treatment is even necessary [68]. An example of a promising investigation is radio-labeled antibodies against carbonic anhydrase-IX with positron emission tomography which has excellent positive and negative predictive values for clear cell phenotype [69].

 Another imaging modality which has already gained widespread acceptance is the use of intraoperative 2D laparoscopic ultrasound to delineate tumor, normal parenchyma, collecting system, and vasculature in an accurate “real-time” fashion [70]. Studies have suggested that intraoperative ultrasound reduces time to hilar dissection and alters clamping approach during LPN [71]. However, as with other forms of ultrasound, it is limited by its dependence on the performance and interpretation [70].

 Another interesting area of current investigation is the use of augmented reality navigation systems, which fuse the anatomic detail of preoperative 3D imaging of CT or MRI with the real-time information of 2D intraoperative ultrasound [72]. Augmented reality imaging can allow the synchronous viewing of the real-time ultrasound image with the corresponding tomogram slice side by side [66].

 Similarly, prototypes of predictive surgical navigation systems are being explored, which have the potential to guide the dissection trajectory for safe and accurate tumor excision in PN [66]. Similar to GPS systems in vehicles, predictive surgical navigational systems use the combination of preoperative 3D imaging with intraoperative ultrasound to correlate the tip of surgical instruments to the surgical target (i.e., tumor) and may assist in determining the surgical anatomy beyond what is directly visible to the surgeon [66]. A color-coded zonal navigational system has been developed, which correlates to the distance from the target [66]. The surgical target appears red on the screen, while the margin within 5 mm of the target appears yellow, 5–10 mm from the target appears as a zone of green, and distances greater than 10 mm from the edge of the target are blue [66]. This technology is still in its infancy but may have a greater role with the increasing utilization of RALPN [66].

## 6. Renal Ischemia

 Several studies suggest a strong correlation between ischemic time and loss of renal function [73, 74]. This has led to the concept that every minute of ischemia counts [75]. Various strategies have been pursued in an effort to reduce the impact of ischemia on renal function [73].

 Recently, the traditionally held belief that ischemic time was the most important determinate of ultimate renal function has been challenged. When factoring in the percent of normal parenchyma preserved, ischemic time was no longer an independent risk factor [76]. The authors of that study believe that ischemic time is likely just a surrogate for the complexity of resection, as there is strong correlation with longer ischemic time with less preservation of parenchyma [76]. The authors also argue that, although ischemic time is predictive of acute kidney injury, it is not predictive of long-term renal function after PN [76]. The authors conclude that the quantity and quality of parenchyma preserved are much more important than duration of ischemic time [76]. Thus, efforts to minimize ischemia should not jeopardize the preservation of as much parenchyma as possible, nor should ischemia take precedence over cancer control steps. However, this does not mean that ischemic time should be ignored. It still remains an important consideration, just not the most important.

During LPN, renal ischemia is typically achieved by clamping of the renal vessels with internal bulldog clamps or exteriorized handheld satinsky clamp [73]. The use of a vessel loop for as a tourniquet can also obtain vascular control [77]. Selective clamping of only the renal artery has been tried [78]; however, high pneumoperitoneum pressure may minimize any benefit by compressing the renal vein [79]. Selective clamping of only the segmental artery that supplies the area of resection has also been reported [80].

 Several strategies have been described to minimize the duration of ischemic time during PN. Early unclamping after tumor resection allows for a bloodless field during tumor resection but minimizes ischemic time during renorrhaphy [81, 82]. Similarly, several “no clamp” techniques have been described, initially reserved for exophytic polar lesions; however, with increasing comfort with LPN, more complex masses are being resected “off clamp” [83]. To aid in minimizing blood loss while “off clamp,” the use of bipolar or ultrasonic-based sealing devices for tumor excision has been used with minimal impact on interpreting margin status [84]. Focal radiofrequency coagulation prior to resection with the Habib 4 also allows for hemostasis without clamping, although cautery artifact can negatively impact examination of the margins [85, 86]. Induced hypotension has also been used in an effort to limit hemorrhage while resecting without clamping [83].

 Although many have reported success in LPN without the need for hilar clamping, one study that used “on-demand” clamping only in the case of excessive bleeding found their reduced ischemic time to be associated with higher rates of blood transfusion and the need for conversion to open for excessive bleeding [87].

Another ischemia minimizing strategy that avoids hilar clamping is the use of parenchyma compression for selective ischemia [88]. This technique is particularly good for polar lesions and exophytic lesions [88]. During HAL PN compression of renal parenchyma near the lesion can be achieved by the surgeon's hand [55]. During pure laparoscopic approaches, a Simon Renal Pole Clamp can be used [88].

 The use of renal cooling is another common strategy for reducing renal functional decline in LPN that has long been a part of an OPN [89–91]. On retrospective analysis, warm and cold ischemia have similar functional outcomes, despite significant longer ischemic time with cold [73, 76]. The longer ischemic time in cold is likely multifactorial, including a selection bias for more difficult reconstructions, the cumbersomeness of hypothemia, and perhaps less sense of urgency for the surgeon [73]. Cold ischemia can be achieved several different ways, such as, surface hypothermia by chilled solutions into a laparoscopic endocatch bag [92], retrograde instillation of cold perfusate [93], or intra-arterial infusion of cold perfusate [94].

Pharmacologic renoprotective measures have also been used to reduce the impact of ischemia. Intravenous mannitol has long been used as an osmotic diuretic and potential free radical scavenger [95]. Similarly, furosemide has been used to promote diuresis after unclamping of the renal vessels [73]. Future therapies might focus on preconditioning the kidney to activate hypoxia-inducible factors that may minimize ischemia injury [96].

## 7. Hemostasis of Tumor Bed and Closure of Collecting System

Suturing is the most effective means of hemostasis and preventing urinary leak; however it is challenging and time-consuming [97, 98]. Several advances in laparoscopy have been applied to LPN to make suturing more practical. The utilization of Hem-o-lok clips and Lapra-Ty clips to replace some of the knots allows for a tight closure with suture that is efficient and secure [98, 99]. For similar reasons the use of barbed suture (Quill or V-Loc) is increasing in popularity for renorrhaphy [97].

 The use of hemostatic agents has allowed an expansion of laparoscopic surgery for increasingly complex resections and reconstructions [100]. It is difficult to compare results of studies on hemostatic agents because of the lack of standardization in controls and because of confounding by the use of multiple agents to achieve hemostasis [100]. Fibrin sealants allow rapid clot formation when applied to a bloodless surgical field and may induce fibroblast migration contributing to a water-tight seal [101, 102]. Gill et al. have shown another sealant, Floseal (human thrombin and bovine gelatin), to reduce complications and hemorrhagic events [103]. Floseal also tends to swell after application, which may provide an additional benefit of mechanical tamponade [100].

 Polyethylene glycol-based sealants and albumin-gluteraldehyde-based sealants undergo covalent polymerization, which can reduce bleeding and urinary leaks during PN [100, 104–106]. Albumin-glutaraldehyde adhesives have even been used alone successfully in “sutureless” PN on select tumors, leading to reduced ischemic time and improved parenchymal preservation [105].

 Additionally, the use of mechanical hemostatic materials, such as oxidized cellulose, provides a mechanical tamponade and a surface for platelet adhesion [100].

As previously mentioned, many studies use a combination of hemostatic methods. The combination of Floseal with rolled bolster has been shown to be most effective for deep resection beds [107].

## 8. Expanding Feasible in Laparoscopic Partial Nephrectomy

 For the reasons previously described, the current AUA guidelines recommend that stage 1 renal masses be treated with PN over RN whenever feasible or advisable as judged by the treating surgeon [3]. Clearly, the term “feasible” is subjective. Furthermore, studies show that PN remains underutilized [108–111], which is likely even more true for minimally invasive approaches [112]. As the aforementioned advances in LPN continue to gain widespread acceptance, it is likely that more tumors will be treated by LPN in the future. Similarly, although current literature on LPN largely reflects the experience of skilled laparoscopic surgeons at centers of excellence, the previously described advances may narrow the proficiency gap to allow LPN to be performed routinely in community settings.

However, it is clear that greater objectivity is needed in describing which tumors are “feasible” for nephron sparing and minimally invasive approaches. Several descriptive systems, such as RENAL score, PADUA score, and C-index, use cross-sectional imaging to add an objective component to the assessment of renal masses [113–115]. The RENAL score and PADUA are similar as they both involve assigning points based on various tumor characteristics [113, 114]. The C-index differs slightly in that it only looks at tumor size and proximity to the kidney's center [115]. The various nephrometry scores have been shown to correlate with ischemic time, perioperative complications, and postoperative estimated GFR [116–118]. These systems provide an objective indicator of tumor complexity allowing for more accurate comparisons of outcomes and practice patterns [119].

## 9. Conclusion

 PN is the standard of treatment for renal tumors due tor the preservation of long-term renal function compared to RN. However, PN currently remains underutilized. LPN offers several benefits over OPN but is more technically challenging and associated with a higher rate of perioperative complications. However, the advances in laparoscopic approaches, imaging modalities, ischemic mitigating strategies, renorrhaphy techniques, and hemostatic agents described previously will likely allow increasingly more complex renal tumors to be amenable to LPN.

## References

[1] Volpe A, Cadeddu JA, Cestari A (2011). Contemporary management of small renal masses. *European Urology*.

[2] Ljungberg B, Cowan NC, Hanbury DC (2010). EAU guidelines on renal cell carcinoma: the 2010 update. *European Urology*.

[3] Campbell SC, Novick AC, Belldegrun A (2009). Guideline for management of the clinical T1 renal mass. *The Journal of Urology*.

[4] Andonian S, Janetschek G, Lee BR (2008). Laparoscopic partial nephrectomy: an update on contemporary issues. *Urologic Clinics of North America*.

[5] Peycelon M, Hupertan V, Comperat E (2009). Long-term outcomes after nephron sparing surgery for renal cell carcinoma larger than 4 cm. *The Journal of Urology*.

[6] Crépel M, Jeldres C, Perrotte P (2010). Nephron-sparing surgery is equally effective to radical nephrectomy for T1BN0M0 renal cell carcinoma: a population-based assessment. *Urology*.

[7] Simmons MN, Weight CJ, Gill IS (2009). Laparoscopic radical versus partial nephrectomy for tumors >4 cm: intermediate-term oncologic and functional outcomes. *Urology*.

[8] Becker F, Siemer S, Hack M, Humke U, Ziegler M, Stöckle M (2006). Excellent long-term cancer control with elective nephron-sparing surgery for selected renal cell carcinomas measuring more than 4 cm. *European Urology*.

[9] Leibovich BC, Blute ML, Cheville JC, Lohse CM, Weaver AL, Zincke H (2004). Nephron sparing surgery for appropriately selected renal cell carcinoma between 4 and 7 cm results in outcome similar to radical nephrectomy. *The Journal of Urology*.

[10] Medina-Polo J, Romero-Otero J, Rodríguez-Antolín A (2011). Can partial nephrectomy preserve renal function and modify survival in comparison with radical nephrectomy?. *Scandinavian Journal of Urology and Nephrology*.

[11] Matin SF, Gill IS, Worley S, Novick AC (2002). Outcome of laparoscopic radical and open partial nephrectomy for the sporadic 4 cm. or less renal tumor with a normal contralateral kidney. *The Journal of Urology*.

[12] Becker F, Siemer S, Humke U, Hack M, Ziegler M, Stöckle M (2006). Elective nephron sparing surgery should become standard treatment for small unilateral renal cell carcinoma: long-term survival data of 216 patients. *European Urology*.

[13] Fergany AF, Hafez KS, Novick AC (2000). Long-term results of nephron sparing surgery for localized renal cell carcinoma: 10-year followup. *The Journal of Urology*.

[14] Weight CJ, Lieser G, Larson BT (2010). Partial nephrectomy is associated with improved overall survival compared to radical nephrectomy in patients with unanticipated benign renal tumours. *European Urology*.

[15] Thompson RH, Boorjian SA, Lohse CM (2008). Radical nephrectomy for pT1a renal masses may be associated with decreased overall survival compared with partial nephrectomy. *The Journal of Urology*.

[16] Huang WC, Elkin EB, Levey AS, Jang TL, Russo P (2009). Partial nephrectomy versus radical nephrectomy in patients with small renal tumors—is there a difference in mortality and cardiovascular outcomes?. *The Journal of Urology*.

[17] Weight CJ, Larson BT, Fergany AF (2010). Nephrectomy induced chronic renal insufficiency is associated with increased risk of cardiovascular death and death from any cause in patients with localized cT1b renal masses. *The Journal of Urology*.

[18] Van Poppel H, Da Pozzo L, Albrecht W (2011). A prospective, randomised EORTC intergroup phase 3 study comparing the oncologic outcome of elective nephron-sparing surgery and radical nephrectomy for low-stage renal cell carcinoma. *European Urology*.

[19] Bernhard JC, Pantuck AJ, Wallerand H (2010). Predictive factors for ipsilateral recurrence after nephron-sparing surgery in renal cell carcinoma. *European Urology*.

[20] Singer EA, Bratslavsky G (2010). Management of locally recurrent kidney cancer. *Current Urology Reports*.

[21] Permpongkosol S, Colombo JR, Gill IS, Kavoussi LR (2006). Positive surgical parenchymal margin after laparoscopic partial nephrectomy for renal cell carcinoma: oncological outcomes. *The Journal of Urology*.

[22] Bensalah K, Pantuck AJ, Rioux-Leclercq N (2010). Positive surgical margin appears to have negligible impact on survival of renal cell carcinomas treated by nephron-sparing surgery. *European Urology*.

[23] Timsit MO, Bazin JP, Thiounn N (2006). Prospective study of safety margins in partial nephrectomy: intraoperative assessment and contribution of frozen section analysis. *Urology*.

[24] Kletscher BA, Qian J, Bostwick DG, Andrews PE, Zincke H (1995). Prospective analysis of multifocality in renal cell carcinoma: influence of histological pattern, grade, number, size, volume and deoxyribonucleic acid ploidy. *The Journal of Urology*.

[25] Bani-Hani AH, Leibovich BC, Lohse CM, Cheville JC, Zincke H, Blute ML (2005). Associations with contralateral recurrence following nephrectomy for renal cell carcinoma using a cohort of 2,352 patients. *The Journal of Urology*.

[26] Russo P, Huang W (2008). The medical and oncological rationale for partial nephrectomy for the treatment of T1 renal cortical tumors. *Urologic Clinics of North America*.

[27] Fehrman-Ekholm I, Dunér F, Brink B, Tydén G, Elinder CG (2001). No evidence of accelerated loss of kidney function in living kidney donors: results from a cross-sectional follow-up. *Transplantation*.

[28] Goldfarb DA, Matin SF, Braun WE (2001). Renal outcome 25 years after donor nephrectomy. *The Journal of Urology*.

[29] Chow WH, Devesa SS, Warren JL, Fraumeni JF (1999). Rising incidence of renal cell cancer in the United States. *Journal of the American Medical Association*.

[30] Hollingsworth JM, Miller DC, Daignault S, Hollenbeck BK (2006). Rising incidence of small renal masses: a need to reassess treatment effect. *Journal of the National Cancer Institute*.

[31] Huang WC, Levey AS, Serio AM (2006). Chronic kidney disease after nephrectomy in patients with renal cortical tumours: a retrospective cohort study. *The Lancet Oncology*.

[32] Bijol V, Mendez GP, Hurwitz S, Rennke HG, Nosé V (2006). Evaluation of the nonneoplastic pathology in tumor nephrectomy specimens: predicting the risk of progressive renal failure. *American Journal of Surgical Pathology*.

[33] Sarnak MJ, Levey AS, Schoolwerth AC (2003). Kidney disease as a risk factor for development of cardiovascular disease: a statement from the American Heart Association Councils on Kidney in Cardiovascular Disease, High Blood Pressure Research, Clinical Cardiology, and Epidemiology and Prevention. *Hypertension*.

[34] Sarnak MJ (2003). Cardiovascular complications in chronic kidney disease. *American Journal of Kidney Diseases*.

[35] Go AS, Chertow GM, Fan D, McCulloch CE, Hsu CY (2004). Chronic kidney disease and the risks of death, cardiovascular events, and hospitalization. *The New England Journal of Medicine*.

[36] Shlipak MG, Fried LF, Cushman M (2005). Cardiovascular mortality risk in chronic kidney disease: comparison of traditional and novel risk factors. *Journal of the American Medical Association*.

[37] Lau WKO, Blute ML, Weaver AL, Torres VE, Zincke H (2000). Matched comparison of radical nephrectomy vs nephron-sparing surgery in patients with unilateral renal cell carcinoma and a normal contralateral kidney. *Mayo Clinic Proceedings*.

[38] McKiernan J, Simmons R, Katz J, Russo P (2002). Natural history of chronic renal insufficiency after partial and radical nephrectomy. *Urology*.

[39] Rini BI (2010). New strategies in kidney cancer: therapeutic advances through understanding the molecular basis of response and resistance. *Clinical Cancer Research*.

[40] Turna B, Aron M, Gill IS (2008). Expanding indications for laparoscopic partial nephrectomy. *Urology*.

[41] Allaf ME, Bhayani SB, Rogers C (2004). Laparoscopic partial nephrectomy: evaluation of long-term oncological outcome. *The Journal of Urology*.

[42] Seifman BD, Hollenbeck BK, Wolf JS (2004). Laparoscopic nephron-sparing surgery for a renal mass: 1-year minimum follow-up. *Journal of Endourology*.

[43] Gill IS, Kavoussi LR, Lane BR (2007). Comparison of 1,800 laparoscopic and open partial nephrectomies for single renal tumors. *The Journal of Urology*.

[44] Gong EM, Orvieto MA, Zorn KC, Lucioni A, Steinberg GD, Shalhav AL (2008). Comparison of laparoscopic and open partial nephrectomy in clinical T 1a renal tumors. *Journal of Endourology*.

[45] Lotan Y, Cadeddu JA (2005). A cost comparison of nephron-sparing surgical techniques for renal tumour. *BJU International*.

[46] Russo P (2010). Partial nephrectomy for renal cancer (part II): the impact of renal ischaemia, patient preparation, surgical approaches, management of complications and utilization. *BJU International*.

[47] Chughtai B, Abraham C, Finn D, Rosenberg S, Yarlagadda B, Perrotti M (2008). Fast track open partial nephrectomy: reduced postoperative length of stay with a goal-directed pathway does not compromise outcome. *Advances in Urology*.

[48] Sprenkle PC, Power N, Ghoneim T (2012). Comparison of open and minimally invasive partial nephrectomy for renal tumors 4–7 centimeters. *European Urology*.

[49] Ramani AP, Desai MM, Steinberg AP (2005). Complications of laparoscopic partial nephrectomy in 200 cases. *The Journal of Urology*.

[50] Lane BR, Novick AC, Babineau D, Fergany AF, Kaouk JH, Gill IS (2008). Comparison of laparoscopic and open partial nephrectomy for tumor in a solitary kidney. *The Journal of Urology*.

[51] Gill IS, Desai MM, Kaouk JH (2002). Laparoscopic partial nephrectomy for renal tumor: duplicating open surgical techniques. *The Journal of Urology*.

[52] Pierorazio PM, Patel HD, Feng T, Yohannan J, Hyams ES, Allaf ME (2011). Robotic-assisted versus traditional laparoscopic partial nephrectomy: comparison of outcomes and evaluation of learning curve. *Urology*.

[53] Kapoor A (2009). Laparoscopic partial nephrectomy: a challenging oraration with a steep learning curve. *Journal of the Canadian Urological Association*.

[54] Lane BR, Chen H, Morrow M, Anema JG, Kahnoski RJ (2011). Increasing use of kidney sparing approaches for localized renal tumors in a community based health system: impact on renal functional outcomes. *The Journal of Urology*.

[55] Strup S, Garrett J, Gomella L, Rowland R (2005). Laparoscopic partial nephrectomy: hand-assisted technique. *Journal of Endourology*.

[56] Gaston KE, Moore DT, Pruthi RS (2004). Hand-assisted laparoscopic nephrectomy: prospective evaluation of the learning curve. *The Journal of Urology*.

[57] Deane LA, Lee HJ, Box GN (2008). Robotic versus standard laparoscopic partial/wedge nephrectomy: a comparison of intraoperative and perioperative results from a single institution. *Journal of Endourology*.

[58] Benway BM, Bhayani SB, Rogers CG (2010). Robot-assisted partial nephrectomy: an international experience. *European Urology*.

[59] Gautam G, Benway BM, Bhayani SB, Zorn KC (2009). Robot-assisted partial nephrectomy: current perspectives and future prospects. *Urology*.

[60] Benway BM, Bhayani SB, Rogers CG (2009). Robot assisted partial nephrectomy versus laparoscopic partial nephrectomy for renal tumors: a multi-institutional analysis of perioperative outcomes. *The Journal of Urology*.

[61] Hyams E, Pierorazio P, Mullins JK, Ward M, Allaf M A comparative cost analysis of robot-assisted versus traditional laparoscopic partial nephrectomy.

[62] Mir SA, Cadeddu JA, Sleeper JP, Lotan Y (2011). Cost comparison of robotic, laparoscopic, and open partial nephrectomy. *Journal of Endourology*.

[63] Lotan Y (2010). Economics of robotics in urology. *Current Opinion in Urology*.

[64] Desai MM, Berger AK, Brandina R (2009). Laparoendoscopic single-site surgery: initial hundred patients. *Urology*.

[65] White MA, Haber GP, Autorino R (2010). Robotic laparoendoscopic single-site surgery. *BJU International*.

[66] Ukimura O, Gill IS (2009). Image-fusion, augmented reality, and predictive surgical navigation. *Urologic Clinics of North America*.

[67] Coll DM, Uzzo RG, Herts BR, Davros WJ, Wirth SL, Novick AC (1999). 3-Dimensional volume rendered computerized tomography for preoperative evaluation and intraoperative treatment of patients undergoing nephron sparing surgery. *The Journal of Urology*.

[68] Krajewski KM, Giardino AA, Zukotynski K, Van den Abbeele AD, Pedrosa I (2011). Imaging in renal cell carcinoma. *Hematology/Oncology Clinics of North America*.

[69] Divgi CR, Pandit-Taskar N, Jungbluth AA (2007). Preoperative characterisation of clear-cell renal carcinoma using iodine-124-labelled antibody chimeric G250 (124I-cG250) and PET in patients with renal masses: a phase I trial. *The Lancet Oncology*.

[70] Secil M, Elibol C, Aslan G (2011). Role of intraoperative US in the decision for radical or partial nephrectomy. *Radiology*.

[71] Hyams ES, Perlmutter M, Stifelman MD (2011). A prospective evaluation of the utility of laparoscopic Doppler technology during minimally invasive partial nephrectomy. *Urology*.

[72] Ukimura O (2010). Image-guided surgery in minimally invasive urology. *Current Opinion in Urology*.

[73] Becker F, Van Poppel H, Hakenberg OW (2009). Assessing the impact of ischaemia time during partial nephrectomy. *European Urology*.

[74] Yossepowitch O, Eggener SE, Serio A (2006). Temporary renal ischemia during nephron sparing surgery is associated with short-term but not long-term impairment in renal function. *The Journal of Urology*.

[75] Thompson RH, Lane BR, Lohse CM (2010). Every minute counts when the renal hilum is clamped during partial nephrectomy. *European Urology*.

[76] Lane BR, Russo P, Uzzo RG (2010). Comparison of cold and warm ischemia during partial nephrectomy in 660 solitary kidneys reveals predominant role of nonmodifiable factors in determining ultimate renal function. *The Journal of Urology*.

[77] Häcker A, Albadour A, Jauker W (2007). Nephron-sparing surgery for renal tumours: acceleration and facilitation of the laparoscopic technique. *European Urology*.

[78] Gong EM, Zorn KC, Orvieto MA, Lucioni A, Msezane LP, Shalhav AL (2008). Artery-only occlusion may provide superior renal preservation during laparoscopic partial nephrectomy. *Urology*.

[79] Tracy CR, Terrell JD, Francis RP (2010). Characterization of renal ischemia using DLP hyperspectral imaging: a pilot study comparing artery-only occlusion versus artery and vein occlusion. *Journal of Endourology*.

[80] Shao P, Qin C, Yin C (2011). Laparoscopic partial nephrectomy with segmental renal artery clamping: technique and clinical outcomes. *European Urology*.

[81] Baumert H, Ballaro A, Shah N (2007). Reducing warm ischaemia time during laparoscopic partial nephrectomy: a prospective comparison of two renal closure techniques. *European Urology*.

[82] Nguyen MM, Gill IS (2008). Halving ischemia time during laparoscopic partial nephrectomy. *The Journal of Urology*.

[83] Eisenberg MS, Patil MB, Thangathurai D, Gill IS (2011). Innovations in laparoscopic and robotic partial nephrectomy: a novel ‘zero ischemia’ technique. *Current Opinion in Urology*.

[84] Phillips JM, Narula N, Deane LA (2008). Histological evaluation of cold versus hot cutting: clinical impact on margin status for laparoscopic partial nephrectomy. *The Journal of Urology*.

[85] Nadler RB, Perry KT, Smith ND (2009). Hybrid laparoscopic and robotic ultrasound-guided radiofrequency ablation-assisted clampless partial nephrectomy. *Urology*.

[86] Wu SD, Viprakasit DP, Cashy J, Smith ND, Perry KT, Nadler RB (2010). Radiofrequency ablation-assisted robotic laparoscopic partial nephrectomy without renal hilar vessel clamping versus laparoscopic partial nephrectomy: a comparison of perioperative outcomes. *Journal of Endourology*.

[87] Bollens R, Rosenblatt A, Espinoza BP (2007). Laparoscopic partial nephrectomy with “on-demand” clamping reduces warm ischemia time. *European Urology*.

[88] Simon J, Bartsch G, Finter F, Hautmann R, De Petriconi R (2009). Laparoscopic partial nephrectomy with selective control of the renal parenchyma: initial experience with a novel laparoscopic clamp. *BJU International*.

[89] Novick AC (1983). Renal hypothermia: in vivo and ex vivo. *Urologic Clinics of North America*.

[90] Thompson RH, Frank I, Lohse CM (2007). The impact of ischemia time during open nephron sparing surgery on solitary kidneys: a multi-institutional study. *The Journal of Urology*.

[91] Shikanov S, Lifshitz D, Chan AA (2010). Impact of ischemia on renal function after laparoscopic partial nephrectomy: a multicenter study. *The Journal of Urology*.

[92] Gill IS, Abreu SC, Desai MM (2003). Laparoscopic ice slush renal hypothermia for partial nephrectomy: the initial experience. *The Journal of Urology*.

[93] Landman J, Venkatesh R, Lee D (2003). Renal hypothermia achieved by retrograde endoscopic cold saline perfusion: technique and initial clinical application. *Urology*.

[94] Beri A, Lattouf JB, Deambros O (2008). Partial nephrectomy using renal artery perfusion for cold ischemia: functional and oncologic outcomes. *Journal of Endourology*.

[95] Nosowsky EE, Kaufman JJ (1963). The protective action of mannitol in renal artery occlusion. *The Journal of Urology*.

[96] Bernhardt WM, Câmpean V, Kany S (2006). Preconditional activation of hypoxia-inducible factors ameliorates ischemic acute renal failure. *Journal of the American Society of Nephrology*.

[97] Sammon J, Petros F, Sukumar S (2011). Barbed suture for renorrhaphy during robot-assisted partial nephrectomy. *Journal of Endourology*.

[98] Orvieto MA, Chien GW, Laven B, Rapp DE, Sokoloff MH, Shalhav AL (2004). Eliminating knot tying during warm ischemia time for laparoscopic partial nephrectomy. *The Journal of Urology*.

[99] Benway BM, Cabello JM, Figenshau RS, Bhayani SB (2010). Sliding-clip renorrhaphy provides superior closing tension during robot-assisted partial nephrectomy. *Journal of Endourology*.

[100] Wheat JC, Wolf JS (2009). Advances in bioadhesives, tissue sealants, and hemostatic agents. *Urologic Clinics of North America*.

[101] Pruthi RS, Chun J, Richman M (2004). The use of a fibrin tissue sealant during laparoscopic partial nephrectomy. *BJU International*.

[102] Johnston WK, Montgomery JS, Seifman BD, Hollenbeck BK, Wolf JS (2005). Fibrin glue V sutured bolster: lessons learned during 100 laparoscopic partial nephrectomies. *The Journal of Urology*.

[103] Gill IS, Ramani AP, Spaliviero M (2005). Improved hemostasis during laparoscopic partial nephrectomy using gelatin matrix thrombin sealant. *Urology*.

[104] Park EL, Ulreich JB, Scott KM (2004). Evaluation of polyethylene glycol based hydrogel for tissue sealing after laparoscopic partial nephrectomy in a porcine model. *The Journal of Urology*.

[105] Hidas G, Kastin A, Mullerad M, Shental J, Moskovitz B, Nativ O (2006). Sutureless nephron-sparing surgery: use of albumin glutaraldehyde tissue adhesive (BioGlue). *Urology*.

[106] Nadler RB, Loeb S, Rubenstein RA, Vardi IY (2006). Use of BioGlue in laparoscopic partial nephrectomy. *Urology*.

[107] Johnston WK, Kelel KM, Hollenbeck BK, Daignault S, Wolf JS (2006). Acute integrity of closure for partial nephrectomy: comparison of 7 agents in a hypertensive porcine model. *The Journal of Urology*.

[108] Dulabon LM, Lowrance WT, Russo P, Huang WC (2010). Trends in renal tumor surgery delivery within the United States. *Cancer*.

[109] Breau RH, Crispen PL, Jenkins SM, Blute ML, Leibovich BC (2011). Treatment of patients with small renal masses: a survey of the American Urological Association. *The Journal of Urology*.

[110] Hollenbeck BK, Taub DA, Miller DC, Dunn RL, Wei JT (2006). National utilization trends of partial nephrectomy for renal cell carcinoma: a case of underutilization?. *Urology*.

[111] Miller DC, Hollingsworth JM, Hafez KS, Daignault S, Hollenbeck BK (2006). Partial nephrectomy for small renal masses: an emerging quality of care concern?. *The Journal of Urology*.

[112] Miller DC, Wei JT, Dunn RL, Hollenbeck BK (2006). Trends in the diffusion of laparoscopic nephrectomy. *Journal of the American Medical Association*.

[113] Kutikov A, Uzzo RG (2009). The R.E.N.A.L. nephrometry score: a comprehensive standardized system for quantitating renal tumor size, location and depth. *The Journal of Urology*.

[114] Ficarra V, Novara G, Secco S (2009). Preoperative aspects and dimensions used for an anatomical (PADUA) classification of renal tumours in patients who are candidates for nephron-sparing surgery. *European Urology*.

[115] Simmons MN, Ching CB, Samplaski MK, Park CH, Gill IS (2010). Kidney tumor location measurement using the C index method. *The Journal of Urology*.

[116] Bruner B, Breau RH, Lohse CM, Leibovich BC, Blute ML (2011). Renal nephrometry score is associated with urine leak after partial nephrectomy. *BJU International*.

[117] Samplaski MK, Hernandez A, Gill IS, Simmons MN (2010). C-index is associated with functional outcomes after laparoscopic partial nephrectomy. *The Journal of Urology*.

[118] Waldert M, Waalkes S, Klatte T (2010). External validation of the preoperative anatomical classification for prediction of complications related to nephron-sparing surgery. *World Journal of Urology*.

[119] Lieser G, Simmons  MN (2011). Developments in kidney tumor nephrometry. *Postgraduate Medicine*.

